# Lack of Association of Acne Severity with Depression, Anxiety, Stress, and Eating Attitudes: A Cross-Sectional Study

**DOI:** 10.3390/jpm14020133

**Published:** 2024-01-23

**Authors:** Mustafa Karaağaç, Hanife Merve Akça, Ömer Acat

**Affiliations:** 1 Department of Psychiatry, Karaman Training and Research Hospital, 70200 Karaman, Türkiye; 2 Department of Dermatology, Faculty of Medicine, Karamanoglu Mehmetbey University, 70200 Karaman, Türkiye; hanifemerveakca@kmu.edu.tr; 3 Karaman Provincial Health Directorate, 70200 Karaman, Türkiye; omer.acat@saglik.gov.tr

**Keywords:** acne vulgaris, depression, anxiety, stress, eating disorder

## Abstract

Background: this study aimed to investigate the relationship between acne severity and depression, anxiety, stress, and negative eating attitudes in patients with acne vulgaris. Method: This study was conducted with 81 patients with acne vulgaris who applied to the dermatology outpatient clinic of Karaman Training and Research Hospital. The patients were asked to complete a sociodemographic data form, the three-factor nutrition questionnaire (TFEQ-21), and the depression anxiety stress scale (DASS-21). Acne severity was assessed using the global acne grading system (GAGS) by an expert dermatologist. Results: Of the 81 patients, 74.1% were female and the average age of the cohort was 22.86 years. The average body mass index of the patients was 21.78 and the GAGS average score was 24.25. Correlation tests revealed the lack of any relationship between the GAGS score and the DASS-21 and TFEQ-21 scale scores (and their subscales). The DASS-21 depression subscale was correlated with the TFEQ-21 total score, and TFEQ-21 emotional eating and TFEQ-21 uncontrolled eating scores. Additionally, a relationship was identified between the DASS21-stress subscale score and TFEQ-21 uncontrolled Eating and TFEQ-21 total score, as well as between the DASS21-anxiety scale and the TFEQ-21 total score and TFEQ-21 uncontrolled eating subscale score. Conclusions: Although no relationship was found between acne severity and depression, anxiety, or eating disorders, these conditions can increase the risk of eating disorders among acne patients. Therefore, it is critical to take the necessary precautions for the treatment of depression and anxiety disorders in this patient population.

## 1. Introduction

Acne vulgaris is characterized by the presence of comedones, papules, pustules, nodules, and scars resulting from chronic inflammation of the pilosebaceous follicles [1]. An increase in sebum production, shedding of follicular epithelial cells, and the involvement of various bacterial agents are implicated in the pathophysiology of acne vulgaris [2]. Global burden of disease studies have reported the approximate worldwide prevalence of acne at 9.4% [3]. Furthermore, acne is considered to be a risk factor for psychiatric illnesses across all ages [4]. Acne vulgaris is associated with various psychiatric disorders, including anxiety disorders, mood disorders, negative body image, and even suicide attempts [5,6,7]. Acne can significantly affect an individual’s self-esteem, interpersonal relationships, and functionality [8].

In recent years, many studies have investigated factors associated with the severity of acne. Mental health disorders such as depression and anxiety are very frequently diagnosed alongside acne. However, there is a growing question about whether these conditions can actually exacerbate the severity of acne. According to the literature, acne and stress have a bidirectional relationship. Thus, stress can worsen the severity of acne, while problems arising from acne, such as social isolation and lack of self-confidence, can lead to depression [2]. In this context, several studies suggest that an increase in stress levels correlates with a worsening of acne severity in a significant portion of acne patients [9,10]. The severity of acne was reported to increase during high-stress periods, such as during exams among university students [11]. However, another study suggested the lack of a direct relationship between perceived stress levels and acne severity. This might reflect individual differences in coping with stress [12].

Eating disorders are psychiatric conditions characterized by aberrant eating behaviors, including excessive food consumption or restrictive dietary patterns [13]. According to international classification systems, eating disorders can be categorized into subtypes such as anorexia nervosa, bulimia nervosa, and binge-eating disorder [14]. Eating disorders can lead to impaired functioning by affecting an individual’s mental state [13]. Stress is a significant factor that influences eating habits and lifestyle. Some studies have indicated that individuals with high-stress levels commonly exhibit a high energy intake and unhealthy eating patterns [15]. Considering the fact that mental disorders such as depression and anxiety often accompany eating disorders, and considering the high prevalence of depression and anxiety among acne patients, eating disorders are also more likely to be seen in patients with acne [16].

Evidence from the published literature indicates the frequent occurrence of psychiatric disorders in patients with acne vulgaris. However, an analysis of existing studies reveals a lack of sufficient data on the relationship between psychiatric comorbidities, such as eating disorders, and the severity of acne. The current study aims to investigate whether levels of stress, depression, and anxiety, as well as negative eating attitudes, impact the severity of acne vulgaris. Additionally, this study seeks to open up new avenues to enhance the quality of life of individuals affected by acne.

## 2. Method

The current study included patients who sought outpatient care at the Dermatology Clinic of Karaman Training and Research Hospital between September and December 2023. These patients were diagnosed with acne vulgaris following an assessment by a specialist dermatologist. All of the patients included in our study had acne distribution either on the face or on both the face and body. The participants were informed about the details of the study and provided written and verbal consent. Individuals with existing psychiatric disorders or undergoing psychiatric treatment, those with chronic systemic diseases or a history of malignancy, individuals who had received acne treatment in the last month, those using medication that could exacerbate acne, pregnant or lactating women, those who did not volunteer to participate in the study, and individuals who filled out the scales incompletely were excluded from this study. Furthermore, the responses provided by the patients in the sociodemographic data form were verified by reviewing their electronic records. Disease severity was assessed using the global acne grading system (GAGS) by a dermatologist specializing in the field. The patients were asked to complete the sociodemographic data form, the three-factor eating questionnaire (TFEQ-21), and the depression anxiety stress scale-21 (DASS-21). The average time for completing these forms was approximately 15 min. After excluding patients who met the exclusion criteria, data from 81 patients who completed the scales were evaluated using different statistical tools.

## 3. Data Collection

Sociodemographic Data Form: a semi-structured sociodemographic data form that was prepared by the researchers was used to obtain the age, gender, educational status, employment status, any additional physical illnesses, tobacco–alcohol–substance use, height, weight, body mass index (BMI), and changes in eating quantity for psychological relief (yes/no) of the study participants.

Three-Factor Eating Questionnaire (TFEQ-21): The TFEQ-21 measures the levels of conscious dietary restraint, uncontrolled eating, and changes in eating behaviors related to emotional states [17]. The questionnaire, translated into Turkish as the “Üç Faktörlü Beslenme Anketi”, has undergone a validity and reliability study by Karakuş et al. [18]. The questionnaire was found to demonstrate structural validity. The reliability analysis indicated that the internal consistency of the items was reasonably reliable for the measurement of dietary habits. The questionnaire consists of 21 items answered on a Likert scale ranging from 1 = definitely false, 2 = mostly false, 3 = mostly true, to 4 = definitely true. The TFEQ comprises three subfactors: uncontrolled eating, emotional eating, and cognitive restraint. A high score indicates a high level of negative eating attitudes [18].

Depression Anxiety Stress Scale (DASS-21): The depression anxiety stress scale (DASS-42) was developed by Lovibond and Lovibond through abbreviation of the original DASS [19]. Scoring 5 or higher on the sub-dimension of depression, 4 or higher on anxiety, and 8 or higher on stress indicates that the individual is experiencing the respective issues. The resulting scores are then classified according to the manual as “normal”, “mild”, “moderate”, “severe”, or “extremely severe”. The psychometric properties of the Turkish version of DASS-21 in both normal and clinical samples were assessed by Sarıçam [20].

Global Acne Grading System (GAGS): The GAGS was developed by Doshi and colleagues and scores the severity of acne based on the distribution of acne on the body and the type of lesions [21]. In the scoring, papules (1), pustules (2), comedones (3), and nodules (4) are evaluated. Scores are assigned for each region of the body and added up to obtain the overall score. The global acne scores of the patients are calculated from 0 to 44. Based on these scores, the acne severity can be categorized as follows: “No acne (0 points), mild acne (1–18 points), moderate acne (19–30 points), severe acne (31–38 points), and very severe acne (>39 points)”.

Ethical approval: The necessary ethical approval was obtained from the Karamanoğlu Mehmetbey University Faculty of Medicine Clinical Research Ethics Committee (IRB no: 05.10.2023-09-2023/08) prior to the start of the study. This study was conducted according to the guidelines of the Declaration of Helsinki.

## 4. Statistical Analysis

Data were analyzed using the IBM SPSS Statistics version 25.0 software package (IBM Corp., Chicago, IL, USA). The normality of continuous data was assessed using q-q plots, skewness, and kurtosis. For continuous variables showing a normal distribution, independent samples *t*-tests were used to determine differences between groups. The Mann–Whitney U test was used for continuous variables that did not show normal distribution and for the analysis of two groups. In cases where the normality assumption was not met, a Spearman correlation test was used to determine the relationship between continuous variables, and a Pearson correlation test was used when the assumption of normality was met. The relationship between acne severity, TFEQ-21, DASS-21 subscale, and total scores was investigated using correlation tests. The interpretation of the correlation coefficient was based on the Spearman rank-order correlation coefficient (weak = 0.01–0.49; moderate = 0.50–0.69; high = 0.70–1.00). Multiple linear regression analysis was conducted to assess the magnitude of the relationship between continuous variables. In regression analyses, autocorrelation was assessed using the Durbin–Watson test, and multicollinearity was evaluated using tolerance and VIF. A statistical significance level of *p* < 0.05 was considered in this study.

## 5. Results

The sociodemographic characteristics of the patients are presented in Table 1 and Table 2. The average age of the patients (*n* = 81) was 22.86 years; 74.1% of the patients were female. Among the participants, 51.9% were high school graduates, while 84% were single. Most of patients reported a lack of use of tobacco or alcohol (86.4%). The average body mass index (BMI) was 21.78, and the mean global acne grading system (GAGS) score was 24.25.

The DASS-21 scores of the patients are shown in Table 3. According to the depression subscale results of the DASS-21, the prevalence of depression among the patients of the current study was 43.2%. Additionally, 58% of the patients had anxiety subscale scores within the normal range, while 75.3% had stress subscale scores within the normal range.

A comparison of the GAGS scores among male and female patients in the current cohort indicated no statistically significant difference in acne severity between male and females (*p* = 0.316, Table 4). However, a comparison of the scores obtained from the DASS-21 and TFEQ-21 scale indicated that the emotional eating subscale scores of the TFEQ-21 of female patients were significantly higher than the male patients (*p* < 0.05). No other statistically significant differences were identified between the scores of the other scales and subscales.

A correlation analysis between the GAGS score of the entire sample and the DASS-21 score, TFEQ-21 scale score, and BMI indicated no statistically significant relationship between the GAGS score and the DASS-21 and TFEQ-21 scale scores (and their subscales) (*p* > 0.05, Table 5). However, a statistically significant positive, weak relationship was identified between the DASS-21 depression subscale and the TFEQ-21 total score (r = 0.260, *p* < 0.01), the TFEQ-21 emotional eating score (r = 0.236, *p* < 0.01), and the TFEQ-21 uncontrolled eating subscale score (r = 0.255, *p* < 0.01). The DASS-21 anxiety scale showed a statistically significant weak relationship with the TFEQ-21 total score (r = 0.400, *p* < 0.01) and the TFEQ-21 uncontrolled eating subscale (r = 0.303, *p* < 0.01). Similarly, the DASS-21 stress scale demonstrated a statistically significant relationship with the TFEQ-21 uncontrolled eating subscale score (r = 0.369, *p* < 0.001) and the TFEQ-21 total score (r = 0.288, *p* < 0.05). A statistically significant relationship was observed between BMI and the TFEQ-21 cognitive restraint subscale score (r = 0.342, *p* < 0.05) and the TFEQ-21 total score (r = 0.231, *p* < 0.05). No statistically significant relationship was identified between BMI and the GAGS or DASS21 scores.

A regression model established between GAGS scores and DASS21 and TFEQ-21 subscales did not exhibit any autocorrelation (Durbin–Watson = 2.139). However, the model did not significantly explain the variation in GAGS scores (F = 0.175(6); Adj. R^2^ = 0.014; *p* = 0.983), accounting for only 1.4% of the variance in GAGS scores (Table 6).

## 6. Discussion

Acne vulgaris, along with eczema and psoriasis, constitutes chronic inflammatory skin conditions that affect individuals of all ages, representing one of the most common reasons for outpatient visits to dermatology clinics [2]. The present study is a cross-sectional investigation focusing on disease severity in patients with acne vulgaris and its relationship with levels of depression, anxiety, stress, and eating attitudes.

Our data indicated that according to the DASS21, the prevalence of depression among the patients examined in the current study was 43.2%. This value is higher compared to the general population (typically averaging 5–10%) [8]. Furthermore, we observed that the frequency of anxiety according to the DASS21 anxiety subscale was 42%. Supporting our data, other studies conducted with patients with acne vulgaris have also reported a high incidence of anxiety and depression [8,22].

Our study demonstrates that acne severity is not associated with depression, anxiety, or stress levels. Although several studies to date have evaluated the relationship between acne severity and depression and anxiety, the results are conflicting. For example, a cross-sectional study conducted in Saudi Arabia reported an association between depression and acne severity, while another study indicated that patients with moderate to severe acne had higher levels of anxiety [23,24]. On the contrary, a study conducted in Turkey did not find any significant difference in anxiety and depression scores between patients with mild and severe acne, supporting the current study [25]. An explanation of the inconsistencies in the results across the different studies is quite challenging. The cross-sectional nature of the studies, differences in the scales used to assess the severity of anxiety and depression (e.g., self-report scales or interviewer-administered scales), and the psychological resilience of the patients may be some of the factors that may account for these differences.

Stress is considered to be a primary factor in exacerbating psychosomatic disorders such as acne vulgaris [2]; various physiological and hormonal changes are known to occur in the body in response to stress [26]. For instance, the release of corticotropin-releasing hormone (CRH) from the hypothalamus, which activates the hypothalamic–pituitary–adrenal (HPA) axis, may trigger the formation of acne lesions [2]. Furthermore, CRH receptor antagonists were reported to be beneficial in the treatment of acne, underscoring the potential significance of this pathway [2]. Most sixth-year medical students in Australia reportedly considered stress as the most significant factor exacerbating acne severity [27]. In a similar vein, a multitude of studies have consistently indicated a correlation between the severity of acne and levels of stress [10,28]. However, contrary to the published data, we could not identify a significant relationship between stress levels and acne severity. While this discrepancy is challenging to explain, it is crucial to consider that some of the studies mentioned above were explicitly conducted on university students experiencing intense stress, particularly during exam periods, which could have influenced the study outcomes. Additionally, in some studies, assessments were not conducted using scales with established psychometric validity. On the other hand, similar to our findings, one previous study has demonstrated the lack of an association between acne severity and stress intensity [12], which was attributed to individual variations in stress tolerance.

Eating disorders are among the psychiatric illnesses that have a severe impact on the quality of life of affected individuals [29]. In a manner akin to acne vulgaris, eating disorders are more prevalent among adolescents, frequently co-occurring with secondary depression and anxiety disorders [30,31]. In recent years, there has been a growing interest worldwide in acne vulgaris and eating disorders. For instance, a recent study by Öner et al. reported that patients with acne vulgaris exhibited more negative eating attitudes compared to healthy controls, albeit reporting no correlation between acne severity and negative eating attitudes [16]. We also could not identify a significant correlation between acne severity and scores of the TFEQ-21 scale and its subscales. Additionally, while eating disorders are generally more prevalent in females, in the current study, we did not identify significant differences in TFEQ-21 scale and subscale scores between males and females, except for TFEQ-21 emotional eating. Although it is difficult to explain why the outcomes of Öner et al. [16] are different from the current study, variations in the designs of these studies may account for this discrepancy. Additionally, the fact that most of our sample consisted of women makes it difficult to draw definitive conclusions regarding the prevalence of eating disorders among female patients with acne.

Anxiety, stress, and depression are well-known predisposing factors for the development of eating disorders in individuals. For instance, university students with increased stress levels had increased negative eating attitudes. Similarly, medical students with high levels of stress were at a higher risk of developing eating disorders [32]. We observed in the current study a significant correlation between the DASS21 stress subscale and both the TFEQ-21 uncontrolled eating score and the TFEQ-21 total score. This suggests that individuals experiencing heightened stress prefer fast food and high-calorie dietary choices over healthier alternatives [33]. Based on our findings, it can be inferred that the coexistence of depression and anxiety in acne patients may contribute to the development of eating disorders. Similarly, the presence of depression and anxiety disorders in adolescents and young adults was shown to lead to a four-fold increase in the likelihood of developing eating disorders [34]. Emotional eating might serve as an indicator of difficulties in emotional regulation associated with depression. Some individuals may resort to emotional eating as a means of coping with stress and negative emotions [35]. Thus, the presence of depression and anxiety disorders in patients with acne vulgaris may necessitate close monitoring and early interventions due to the potential development of eating disorders.

One parameter closely associated with eating disorders is body mass index (BMI). Öner et al. demonstrated a correlation between negative eating attitudes and BMI among acne patients [16]. Supporting these findings, we identified a significant relationship between TFEQ-21 total score and TFEQ-21 cognitive restraint. Furthermore, various studies have provided evidence of an association between BMI and emotional eating [36,37,38]. In the realm of eating disorders, BMI is often used as one of several indicators to assess the severity of the disorder and monitor changes in weight over time. However, it is important to note that individuals with eating disorders may not always present with extreme deviations in BMI. Furthermore, the relationship between BMI and eating disorders is complex and multifaceted. While low BMI is a common feature of anorexia nervosa, other eating disorders such as bulimia nervosa or binge-eating disorder may be associated with normal or higher BMI. In light of these findings, it can be suggested that early intervention for eating disorders may be effective in addressing various metabolic issues in acne patients.

In conclusion, although our study has revealed significant findings regarding the relationship between acne vulgaris and depression, anxiety, stress, and eating attitudes, caution is warranted in interpreting these results. Firstly, it is important to acknowledge that our study had a relatively small sample size and was conducted at a single center, which may limit the generalizability of our findings to broader populations. Moreover, the similarity in sociodemographic characteristics among our participants might restrict the applicability of our results to more diverse populations. Secondly, we did not include a healthy control group in the current study, precluding a comparison of the data with a control group. Thirdly, the patients were not examined by a specialist psychiatrist; therefore, undiagnosed psychiatric illnesses among these patients may not have been accounted for but may have influenced the study results. Fourth, in our study, despite the prior validation and reliability assessments of the self-report measures used, we cannot ignore the potential for response bias. Therefore, the use of interviewer-administered scales in future studies could mitigate this issue and provide a more accurate representation of the participants’ responses. Finally, due to the cross-sectional design of this study, it is impossible to make definitive conclusions about a cause-and-effect relationship. Therefore, to address these limitations, future studies with a longitudinal design, larger sample size, and structured interviews with participants should be conducted to validate our results.

## Figures and Tables

**Table 1:** The sociodemographic data of patients with acne vulgaris.

		Frequency	Valid Percent	Cumulative Percent	
Gender	Male	21	25.9	25.9	
	Female	60	74.1	100.0	
Educational level	High School	42	51.9	51.9	
	Middle School	4	4.9	56.8	
	University	34	42.0	98.8	
	Master’s/Ph.D.	1	1.2	100.0	
Employment status	Yes	17	21.0	21.0	
	No	64	79.0	100.0	
Level of Income	Low	16	19.8	19.8	
	Middle	64	79.0	98.8	
	High	1	1.2	100.0	
Marital status	Single	68	84.0	84.0	
	Married	13	16.0	100.0	
Tobacco and/or alcohol use	Neither	70	86.4	86.4	
	Tobacco	11	13.6	100.0	
	Total	81	100.0		
	Mean	Std. Deviation	Median	Q1 (25)	Q3 (75)
Age	22.68	4.86	21.0	19.0	26.0
Weight (kg)	60.10	10.65	59.0	53.0	64.5
BMI	21.78	3.55	21.4	19.4	23.1
Height	166.11	7.74	167.0	160.0	171.0

**Table 2:** GAGS, DASS21, and TFEQ21 scores of patients with acne vulgaris.

	n	Mean	Std. Deviation	Median	Q1 (25)	Q3 (75)
GAGS Score	81	24.25	8.74	24.0	16.0	32.0
DASS21-Depression	81	4.68	4.58	3.0	1.0	6.5
DASS21-Anxiety	81	4.26	3.83	3.0	1.0	7.0
DASS21-Stress	81	5.28	4.25	4.0	2.0	7.5
DASS21_Total	81	14.22	11.31	10.0	5.0	19.0
TFEQ21-Cognitive Restraint	81	10.79	4.24	10.0	7.0	13.5
TFEQ21-Emotional Eating	81	8.86	3.85	7.0	6.0	11.0
TFEQ21-Uncontrolled Eating	81	16.22	5.29	16.0	12.0	20.0
TFEQ21-Total	81	35.88	9.21	36.0	29.0	41.0

**Table 3:** DASS21 subscale scores of patients with acne vulgaris.

		*n*	%
DASS21-Depression	Normal	46	56.8
	Mild	15	18.5
	Moderate	9	11.1
	Severe	4	4.9
	Extremely Severe	7	8.6
DASS21-Anxiety	Normal	47	58.0
	Mild	10	12.3
	Moderate	7	8.6
	Severe	8	9.9
	Extremely Severe	9	11.1
DASS21-Stress	Normal	61	75.3
	Mild	6	7.4
	Moderate	9	11.1
	Severe	2	2.5
	Extremely Severe	3	3.7
	Total	81	100.0

**Table 4:** Comparison of GAGS score with DASS21 and TFEQ-21 scale scores by gender.

			Mean	Std. Deviation	Median	Q1 (25)	Q3 (75)	*p*
GAGS Score	Male	21	25.90	8.36	28	17	34	0.316 *
	Female	60	23.67	8.87	24	16	31	
DASS21-Depression	Male	21	3.90	3.94	3	1	5	0.410 ^Ω^
	Female	60	4.95	4.79	4	1	7	
DASS21-Anxiety	Male	21	3.00	2.41	3	1	5	0.750 *
	Female	60	4.70	4.15	3	1	8	
DASS21-Stress	Male	21	5.05	4.04	4	2	10	0.783 ^Ω^
	Female	60	5.37	4.35	5	2	7	
DASS21-Total	Male	21	11.95	8.85	10	4	19	0.493 ^Ω^
	Female	60	15.02	12.02	12	5	22	
TFEQ21-Cognitive Restraint	Male	21	10.29	2.78	10	8	12	0.940 ^Ω^
	Female	60	10.97	4.65	10	7	14	
TFEQ21-Emotional Eating	Male	21	7.19	1.91	6	6	8	0.014 ^Ω^
	Female	60	9.45	4.19	8	6	11	
TFEQ21-Uncontrolled Eating	Male	21	16.52	5.04	16	13	21	0.764 *
	Female	60	16.12	5.42	16	11	20	
TFEQ21-Total	Male	21	34.00	6.66	33	30	38	0.197 *
	Female	60	36.53	9.91	37	29	43	

* Independent samples *t*-test; ^Ω^ Mann–Whitney U test.

**Table 5:** Correlation results between GAGS score, DASS21, TFEQ21, and BMI.

		DASS21-Depression	DASS21-Anxiety	DASS21-Stress	DASS21-TOT	TFEQ21-Cognitive Restraint	TFEQ21-Emotional Eating	TFEQ21-Uncontrolled Eating	TFEQ21-Total	BMI
GAGS Score	r	0.056 _(b)_	0.071 _(a)_	0.115 _(b)_	0.103 _(b)_	0.053 _(b)_	0.008 _(b)_	0.065 _(a)_	0.089 _(a)_	−0.139 _(b)_
	*p*	0.618	0.528	0.305	0.361	0.639	0.947	0.566	0.431	0.216
DASS21-Depression	r		0.688 ** _(b)_	0.656 ** _(b)_	0.883 ** _(b)_	0.057 _(b)_	0.236 * _(b)_	0.255 * _(b)_	0.260 * _(b)_	−0.042 _(b)_
	*p*		<0.001	<0.001	<0.001	0.616	0.034	0.022	0.019	0.713
DASS21-Anxiety	r			0.651 ** _(b)_	0.851 ** _(b)_	0.135 _(b)_	0.213 _(b)_	0.303 ** _(a)_	0.400 ** _(a)_	−0.025 _(b)_
	*p*			<0.001	<0.001	0.229	0.056	0.006	<0.001	0.827
DASS21-Stress	r				0.891 ** _(b)_	0.008 _(b)_	0.212 _(b)_	0.369 ^**^_(b)_	0.288 ** _(b)_	−0.045 _(b)_
	*p*				<0.001	0.944	0.057	<0.001	0.009	0.690
DASS21-Total	r					0.058 _(b)_	0.246 * _(b)_	0.333 ** _(b)_	0.306 ** _(b)_	−0.057 _(b)_
	*p*					0.608	0.027	0.002	0.006	0.614
TFEQ21-Cognitive Restraint	r						0.183 _(b)_	0.104 _(b)_	0.558 ** _(b)_	0.342 ** _(b)_
	*p*						0.101	0.353	<0.001	0.002
TFEQ21-Emotional Eating	r							0.481 ** _(b)_	0.690 ** _(b)_	0.184 _(b)_
	*p*							<0.001	<0.001	0.099
TFEQ21-Uncontrolled Eating	r								0.798 ** _(a)_	0.003 _(b)_
	*p*								<0.001	0.977
TFEQ21-Total	r									0.231 * _(b)_
	*p*									0.038

(a) Pearson correlation test; (b) Spearman correlation test.* *p* < 0.05, ** *p* < 0.01

**Table 6:** Regression analysis results for GAGS score, DASS21, and TFEQ21 subscales.

						B %95 CI	
	β	Std. Error	t	<0.001	B	Minimum	Maximum
(Constant)		4.174	5.064	0.000	21.136	12.820	29.452
DASS21-Depression	0.032	0.348	0.174	0.863	0.060	−0.633	0.754
DASS21-Anxiety	0.084	0456	0.421	0.675	0.192	−0.717	1.101
DASS21-Stress	−0.102	0.370	−0.567	0.573	−0.209	−0.946	0.527
TFEQ21-Cognitive Restraint	0.042	0.244	0.352	0.726	0.086	−0.400	0.571
TFEQ21-Emotional Eating	0.021	0.313	0.155	0.877	0.049	−0.575	0.673
TFEQ21-Uncontrolled Eating	0.066	0.235	0.461	0.646	0.109	−0.361	0.578

Multiple linear regression analysis.

## Data Availability

The data presented in this study are available on request from the corresponding author.
